# The therapeutic potential of irisin to mitigate the risk of metabolic syndrome in postmenopausal women

**DOI:** 10.3389/frph.2024.1355922

**Published:** 2024-07-08

**Authors:** Rebecca A. Parkin, Andrew J. Murray

**Affiliations:** Department of Physiology, Development and Neuroscience, University of Cambridge, Cambridge, United Kingdom

**Keywords:** menopause, metabolic syndrome, oestrogen, irisin, myokine

## Abstract

Oestradiol withdrawal at menopause predisposes women to metabolic syndrome, a cluster of interrelated conditions including obesity, insulin resistance, dyslipidaemia and hypertension that together confer an increased risk of developing type 2 diabetes mellitus and cardiovascular disease. Hormone replacement therapies are commonly used to treat acute symptoms of the perimenopausal period, and whilst they have been associated with metabolic improvements in many studies, long-term use is considered unviable. Novel approaches are required to mitigate the risk of postmenopausal metabolic syndrome. In 2012, the exercise-inducible myokine irisin was isolated from the skeletal muscle of mice and identified to have anti-obesity and antidiabetic effects *in vivo*. Irisin is now recognised to exert pleiotropic action on cognitive, bone and metabolic health. There is accumulating evidence from *in vitro* and *in vivo* rodent studies that irisin can mitigate each component condition of metabolic syndrome. In postmenopausal women, independent associations have been observed between (a) exercise and plasma irisin concentration and (b) plasma irisin concentration and reduced incidence of metabolic syndrome. To date, however, no study has considered the mechanistic basis by which irisin, whether exercise-induced or exogenously administered, could reduce the incidence or severity of metabolic syndrome in postmenopausal women. This review aims to analyse the literature concerning the metabolic actions of irisin, with a focus on its therapeutic potential for metabolic syndrome driven by a state of oestradiol depletion. It evaluates the practicality of exercise as a therapy and discusses other irisin-based therapeutic strategies that may alleviate postmenopausal metabolic syndrome. Finally, it highlights areas where future research is required to advance knowledge of irisin's biological action such that it could be considered a viable candidate for clinical application.

## 1 Introduction

Women experience natural menopause, clinically defined as 12 months of amenorrhea, between the ages of 45 and 55 years (1). The major endocrinological feature of the peri- and postmenopausal states is 17β-oestradiol and progesterone withdrawal as a consequence of the decline and eventual cessation of ovarian function. In addition to menstrual cycle irregularity, variability in the sex hormone milieu during the perimenopausal period confers acute physiological and psychological symptoms that often subside with time and, where required, may be treated with hormone replacement therapies (HRT) (2). Conversely, sustained postmenopausal sex hormone depletion, particularly that of oestradiol, is present for the remainder of life and is associated with pathophysiological conditions including an increased risk of metabolic syndrome (MetS) (3). MetS is the co-occurrence of insulin resistance (IR), visceral adiposity, atherogenic dyslipidaemia and endothelial dysfunction (4). These distinct yet tightly interrelated conditions are, in turn, associated with an elevated risk of cardiovascular disease (CVD) and type 2 diabetes mellitus (T2DM) by 2- and 5-fold, respectively (5). Numerous studies, a selection of which are summarised in Table 1 (6–8), have identified positive associations between postmenopausal status and the risk and prevalence of MetS or its component conditions, independent of age, ethnicity, and other confounding factors.

**Table 1:** The risk of developing MetS is higher in postmenopausal women compared with premenopausal counterparts. Janssen et al. found that MetS prevalence increases during the perimenopausal period; therefore, the elevation of risk is not exclusive to the postmenopausal state and coincides with the onset of ovarian sex hormone decline, rather than total depletion.

Population	Cohort size	Metabolic syndrome		Reference
		Pre-menopause	Post-menopause	
Iran	940	18.3% prevalence	53.5% prevalence	(6)
South Korea	2,671		1.6-fold risk increase	(7)
USA (SWAN)	949	19.0% prevalence	32.7% prevalence (at final menses)	(8)

SWAN, Study of Women's Health Across the Nation.

Whilst some suggest menopause-associated metabolic risk derives from an increase in the body's testosterone:oestrogen ratio, oestradiol withdrawal alone constitutes the shift towards the proposed testosterone-dominant state (9). Oestradiol depletion is postulated to be the driver of postmenopausal MetS, a theory supported by evidence from studies in cell lines and rodent models, as well as human HRT studies illustrating the oestrogenic contribution to the regulation of metabolism and metabolic risk factors (10). A meta-analysis of 107 randomised controlled trials (RCT) showed short-term oestrogen-only HRT attenuated rates of postmenopausal MetS (11). Long-term oestrogen-only HRT presents a possible therapeutic solution but has significant limitations, including side effects that are intolerable for some women. There is considerable debate about absolute risks, however oestrogen-only formulations are associated with marginally elevated risks of breast and endometrial cancers, thromboembolism, and stroke and are therefore contraindicated in women at increased risk of these conditions. Furthermore, the critical window hypothesis, established in the treatment of acute menopausal cognitive symptoms and proposed to apply to HRT's cardioprotective effects, suggests efficacy decreases as the relative duration between perimenopausal symptom emergence and therapy onset increases (12). The risks and benefits of long-term HRT lack RCT-supported evidence, but current guidance recommends limiting use to 5 years, indicating a perception of detrimental consequences.

The risk of developing MetS post-menopause is not trivial; it poses significant consequences for a woman's quality of life, and wider society through the economic burden of clinically managing component conditions. Critically, MetS is both preventable and reversible. There is a great need to understand how the risk of MetS can be effectively managed in postmenopausal women. Given the purported limitations of HRT, novel therapeutic approaches are essential.

**Figure 1:**
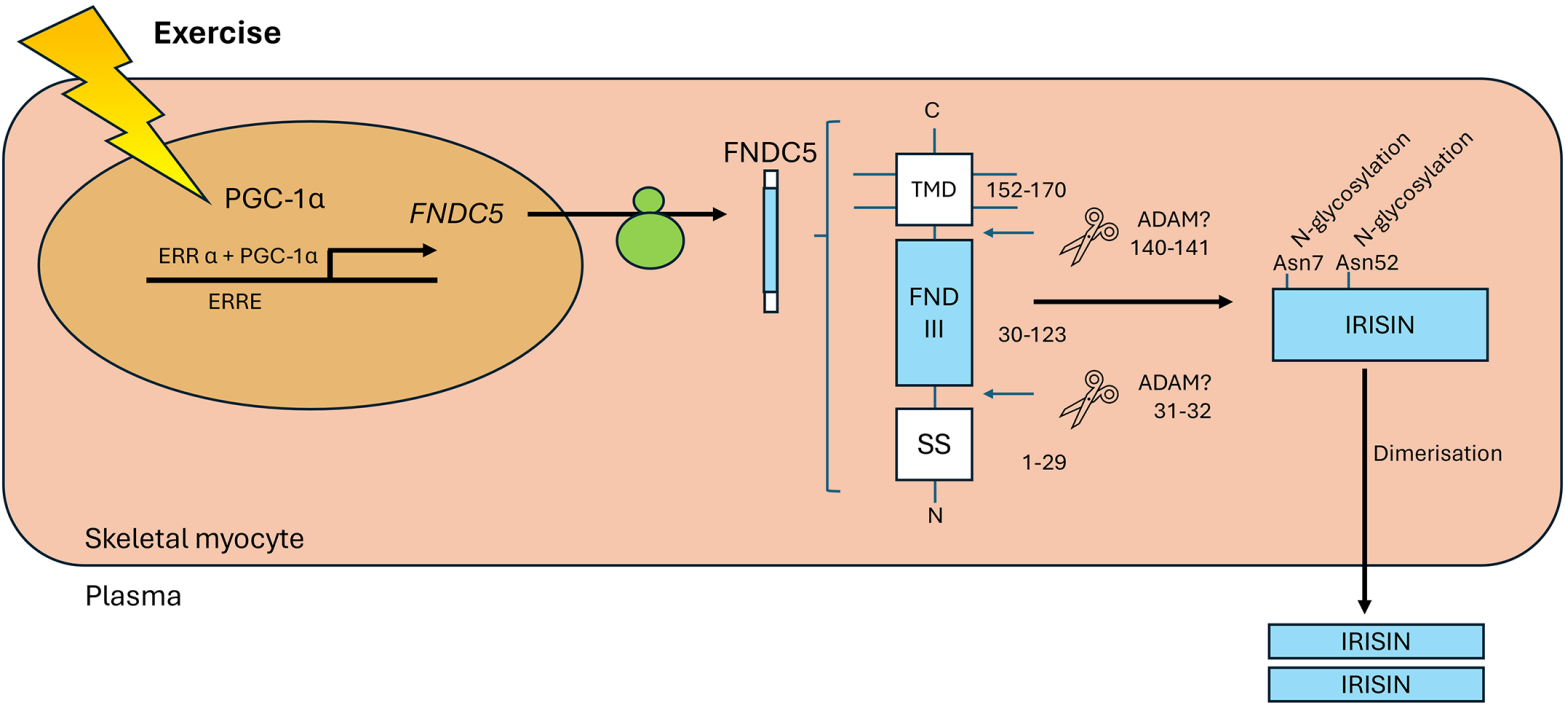
Exercise increases the levels of PGC-1α in myocytes, which interacts with ERRα to increase expression of *FNDC5*. Once trafficked to the sarcolemma, proteases cleave the extracellular portion of FNDC5 at two sites (hypothesised but not confirmed to be by ADAMs, predicted positions indicated). The soluble 112 amino acid peptide product is released to the plasma as a homodimer. Figure adapted from (18) and (19). PGC-1α, peroxisome proliferator-activated receptor γ coactivator-1α; ERRα, oestrogen related receptor α; ERRE, oestrogen related response element; FNDC5, fibronectin type III domain-containing 5; TMD, transmembrane domain; FND III, fibronectin type III domain; SS, signal sequence; ADAM, a disintegrin and metalloproteinase.

The hormone irisin was initially found to be expressed in mouse skeletal muscle and was shown to promote the browning of white adipose tissue, supporting a thermogenic phenotype (13). It is produced by the proteolytic cleavage of the transmembrane glycoprotein, fibronectin type III domain-containing 5 (FNDC5), the expression of which increased in response to exercise in a manner dependent on peroxisome proliferator-activated receptor γ co-activator 1α (PGC-1α). The presence and physiological role of irisin in humans was initially considered controversial, owing to the presence of a non-canonical translational start codon (ATA rather than ATG) in human *FNDC5* (14, 15) and the poor specificity of the antibodies initially used to detect circulating irisin (14). The existence of circulating irisin was however confirmed in humans using a quantitative tandem mass spectrometry approach (16). Irisin is predominantly expressed from its unconventional ATA start codon, and circulating levels increase in response to exercise. Of note, however, irisin levels in human plasma are much lower than those reported in rodents, being ∼3–5 ng/ml in humans, depending on activity status (16), compared with ∼300–600 ng/ml in rats (17). In humans, circulating irisin homodimers are primarily derived from skeletal muscle secretion in response to exercise (Figure 1), although production in multiple tissues has been identified by immunohistochemistry (20). A definitive receptor has not yet been elucidated, although action via αV-containing integrins is proposed in bone and adipose tissue (21).

Beyond its role as a browning agent in adipose tissue, irisin is now recognised to exert pleiotropic actions, as reviewed by Liu et al. (19). Numerous downstream metabolic actions of irisin mirror those of oestradiol. As a recently-recognised hormone, its potential for therapeutic application in scenarios of oestradiol depletion is an intriguing concept that has received little attention. Independent, positive associations between regular exercise and serum irisin concentrations, and negative associations between regular exercise and biochemical markers of MetS have been reported in postmenopausal women with T2DM (22). Despite the absence of a causal link between exercise-induced irisin elevation and the reduction in metabolic risk factors, this study supports the need for further investigation. This review aims to evaluate the potential of irisin, whether exercise-induced or administered as a recombinant protein, as a therapeutic agent for mitigating the risk of MetS that arises secondary to oestradiol withdrawal in postmenopausal women.

## 2 Evidence for the mitigating actions of irisin against components of post-menopausal MetS and related conditions

### 2.1 Adiposity

In the context of metabolic risk, the location of body fat storage is critical. Obese individuals with relatively high gluteofemoral subcutaneous adipose tissue (SAT) mass are often described as having “metabolically healthy” obesity in which insulin sensitivity is largely retained, as opposed to the “metabolically unhealthy” phenotype of greater abdominal SAT and, more significantly, intra-abdominal visceral adipose tissue (VAT) deposition (23). Visceral adiposity is associated with increased cardiometabolic risk, as it confers a state of chronic low-grade inflammation, dysregulated lipid metabolism and an altered adipokine profile. The dysfunctionality of hypertrophically expanded VAT promotes ectopic triglyceride deposition within tissues of non-lipid-storing organs, further exacerbating the metabolically unfavourable state (23). Menopause is associated with increased adiposity, biased towards visceral depots, following the loss of oestrogenic regulation of adipogenesis, lipogenesis and lipolysis (24, 25). HRT can reduce, or even prevent, menopause-associated increases in body weight and central fat distribution (24, 26, 27).

In conjunction with its initial discovery in mice, irisin's ability to reduce diet-induced obesity was described (13). Although irisin acts pleiotropically, direct impacts on adipocyte number and lipid content have been observed. Irisin inhibited adipogenic differentiation and instead promoted an osteogenic programme in de-differentiated preadipocytes with mesenchymal stem cell (MSC) properties (28). Should this occur *in vivo*, this action has potential to confer dual benefits for postmenopausal women by targeting adiposity, relevant to MetS, but also bone health, which also deteriorates upon menopausal oestradiol depletion (1). It is possible that by inhibiting adipogenic commitment, hypertrophic expansion of existing adipose tissues would be promoted by irisin under a state of positive energy balance; however, irisin's ability to enhance energy expenditure (discussed later) may mitigate this risk.

Ma et al. (29) identified additional actions of irisin later in the adipogenic programme. Transfection of 3T3-L1 preadipocytes with plasmids expressing *FNDC5*, or addition of recombinant irisin to differentiation media, reduced relative mRNA and protein expression of the key adipogenic genes encoding peroxisome proliferator-activated receptor γ (PPARγ), CCAAT-enhancer-binding protein α (C/EBPα), and fatty acid binding protein 4 (FABP4), and this was associated with a reduction in mature adipocyte count. Moreover, whilst Wnt signalling is downregulated during adipogenesis the expression of several Wnt isoforms was maintained or upregulated by irisin. The authors noted however that basal endogenous irisin levels were insufficient to attenuate adipogenesis; siRNA knockdown of *FNDC5* reduced Wnt expression but this did not impact mature adipocyte formation, with expression of FABP4, PPARγ, and C/EBPα being unperturbed.

Oestradiol reduces adipocyte capacity for *de novo* lipogenesis by downregulating expression of lipoprotein lipase, fatty acid synthase and acetyl-coenzyme A carboxylase 1 (30). Irisin is not known to modulate adipose tissue lipogenesis, however early *in vitro* work has indicated that, like oestradiol, it upregulates lipolytic enzyme expression. In 3T3-L1 derived adipocyte cell lines, human recombinant irisin increased expression of *PLIN*, *LIPE* and *PNPLA2*, encoding perilipin 1 (PLIN1), hormone sensitive lipase (HSL) and adipose triglyceride lipase (AGTL), respectively, and this corresponded with an elevation in cellular ATP (31). Levels of PLIN1 and AGTL mRNA remained elevated for at least 24 h post-administration, which is potentially promising when considering the duration of irisin's effects following exercise or alternative therapeutic application. Of note, HSL mRNA was only significantly elevated at one timepoint (8 h) following irisin treatment; oestradiol also does not increase HSL protein levels, as it does PLIN1 and ATGL (30, 32). Further work is required to establish whether an oestradiol-like depot specificity that facilitates preferential deposition of SAT exists for irisin. Notably, WAT browning by irisin, (discussed later) has been shown to occur exclusively in SAT (33–35).

High-intensity interval training in postmenopausal women and endurance training in middle-aged/older adults has been associated with increased plasma irisin concentrations and reductions in visceral adiposity (36, 37). It is unknown whether irisin specifically reduces VAT mass, and more generally, delineating the relative contributions of irisin and exercise *per se* to fat loss is challenging. Irrespective of irisin's specific actions, these studies at least highlight that exercise in a range of forms will, to some extent, increase plasma irisin levels and reduce overall obesity.

Oestradiol exerts indirect anti-inflammatory action by limiting VAT deposition and promoting hyperplastic adipose expansion, whereas irisin appears to directly modulate primary inflammatory responses. Irisin administration, albeit at a supraphysiological concentration, caused a dose-dependent attenuation of pro-inflammatory cytokine expression in 3T3-L1 derived adipocytes (38). The macrophage recruitment signal monocyte chemotactic protein 1 (MCP-1) was also downregulated. Later experiments by Xiong et al. (39) showed irisin deficient (*Fndc5*^−/−^), high-fat diet (HFD) fed mice had exacerbated inflammation and increased macrophage recruitment to adipose tissue compared with *Fndc5* competent HFD-fed controls, perhaps reflecting dysregulation of MCP-1 expression. *In vitro*, irisin inhibited lipopolysaccharide induced M1 polarisation in macrophages isolated from mouse peritoneal cavities via an AMP-activated protein kinase (AMPK) dependent mechanism, resulting in a pro- to anti-inflammatory shift in cytokine profile. These effects were also seen in mice *in vivo*, where lentiviral vector-mediated *Fndc5* overexpression in skeletal muscle conferred anti-inflammatory M2 polarisation of adipose tissue-resident macrophages and attenuated IR in obese wild-type and whole-body *Fndc5*^−/−^ mice. Of particular relevance to VAT in humans, *Fndc5*^−/−^ obese mice exhibited adipocyte hypertrophy, highlighting an additional, oestradiol-like facility through which irisin may improve adipose tissue metabolic function in obese individuals, albeit one without a mechanistic basis as yet. The relative contribution of inflammation from macrophages and adipocytes themselves to the insulin-resistant state of *Fndc5*^−/−^ mice is challenging to quantify, since these mice were obese and likely experienced alterations in energy expenditure. Nevertheless, these findings contribute to an understanding of the potential impacts of irisin on MetS, given visceral obesity and its consequences are integral to the condition.

### 2.2 Energy balance

Obesity develops if energy intake chronically exceeds expenditure. A positive energy balance is not a component of MetS *per se* but is a significant driver of obesity. Oestrogenic contribution to energy balance regulation has been demonstrated in mouse models. Ovariectomy (OVX) caused 25% more weight gain in mice compared with sham-operated controls, despite equivalent food consumption (40), suggesting a reduction in energy expenditure. Moreover, if *ad libitum* feeding is permitted, ovariectomised mice exhibit hyperphagia (41). Postmenopausal women have lower resting and active metabolic rates than premenopausal individuals of equivalent body mass index (BMI) (42). Irisin's effects on adipocyte development have been outlined previously; however, its predominant anti-obesity action may lie in the modulation of energy balance, particularly expenditure, a critical contributor to postmenopausal obesity, and by extension MetS.

In mitochondria-dense, multilocular adipocytes of brown adipose tissue (BAT), ATP synthesis is uncoupled from the mitochondrial electron transport chain via proton gradient dissipation through uncoupling protein 1 (UCP1). Both irisin and oestradiol can transdifferentiate white adipocytes to a “beige” phenotype with increased capacity for non-shivering thermogenesis (NST). Irisin was shown to induce the browning of white adipose tissue (WAT) *in vitro* and *in vivo*, characterised by upregulation of *Ucp1*, development of multilocular lipid droplets and an increase in oxygen consumption (13). There remains a lack of consensus on the intracellular signalling pathways that promote the expression of genes required for transdifferentiation.

Early *in vitro* evidence suggests irisin also increases beige adipose mass by promoting “beige adipogenesis”. Irisin binding to transient receptor potential channel 3 (TRPC3) on human adipose-derived MSCs induced direct differentiation to beige adipocytes with elevated UCP1 expression (43). Additionally, irisin interaction with a CD81-integrin αV-β1/β5 complex expressed on the plasma membrane of a CD81^+^ beige preadipocyte subpopulation present in mouse inguinal adipose tissue induced proliferation through focal adhesion kinase signalling (44). Oestrogen receptor α agonism in murine adipocyte stem cells upregulates brown adipogenic genes and drives a multilocular phenotype, highlighting the potential of irisin as a therapeutic agent, specifically in states of oestradiol depletion (45).

An irisin-mediated increase in thermogenic capacity may present a mechanism by which the postmenopausal reduction in energy expenditure, consequent to the loss of oestradiol-mediated disinhibition of sympathetic NST stimulation, might be offset (46). Irisin appears to be a promising agent for targeting energy imbalance, however, there may be a major caveat limiting its therapeutic potential in postmenopausal women. First reported *in vivo* in mice, and since shown in two independent experiments on isolated human adipocytes, irisin was found to only transdifferentiate white adipocytes of SAT, and not VAT (13, 34, 35). Should this depot specificity of irisin action extend to human adipose tissues *in vivo*, it is possible that irisin would be ineffective in limiting and reversing postmenopausal increases in VAT, and higher VAT:SAT mass ratios could thus limit the efficacy of irisin-based therapies to increase energy expenditure. Women do retain SAT mass through menopause, and many individuals accumulate more, particularly in abdominal regions, therefore, tissues susceptible to browning are not entirely absent. These findings give rise to an interesting hypothetical concept, in that irisin may be subject to a “critical window” period, somewhat analogous to HRT, during which WAT thermogenic capacity may be enhanced to restore neutral or negative energy balance before visceral adiposity accelerates. It follows that investigation of other adipose-related conditions improved by irisin, including IR and dyslipidaemia, is needed to establish the existence and impact of any depot-specific action.

Oestradiol is thought to exert anorexigenic action at the hypothalamus by altering neuronal excitability in the paraventricular nucleus and increasing brain-derived neurotrophic factor (BDNF) levels, and thus oestradiol withdrawal would further exacerbate the energy imbalance experienced by postmenopausal women (47). Irisin is proposed to similarly modulate hypothalamic feeding regulation via anorexigenic neurotransmitter upregulation, although whether this translates into behavioural alteration is controversial. Intrahypothalamic irisin injections decreased food consumption in normally-fed but not fasted rats, implying modulation of the normal, unstressed regulatory mechanisms for feeding. Reduced feeding was associated with increased hypothalamic expression of the anorexigenic neurotransmitters cocaine- and amphetamine-regulated transcript (CART) and pro-opiomelanocortin (POMC) (48). Intraperitoneal irisin injection increased hypothalamic mRNA expression of CART, POMC, neuropeptide Y (NPY) and BDNF, but conversely failed to alter body weight and feeding behaviour (49). The authors note that whilst NPY is orectic and may offset the effects of POMC/CART upregulation, it is a central neurotransmitter expressed throughout the brain, therefore upregulation may not specifically impact feeding. The study was conducted over 14 days, which may be an insufficient period for alterations in gene expression to change behaviour. Despite a lack of clarity regarding irisin's precise actions on feeding behaviour, these observations support the once-debated concept that irisin, or at least a secondary factor it regulates, crosses the blood-brain barrier (BBB).

By an undefined molecular pathway, irisin increases central and peripheral tissue BDNF concentrations (18). BDNF, either synthesised within the brain or transported across the BBB from the systemic circulation, has well-established neuroprotective actions, leading to the proposal of a PGC-1α/FNDC5/BDNF axis in the hippocampus that likely exists elsewhere in the brain (50). There is evidence to support an anorexigenic action for BDNF from rodent studies including gene knockouts and peripheral infusions that promoted hyperphagia and hypophagia (51). A relative lack of attention has been devoted to the regulation of feeding behaviour by BDNF at the hypothalamus compared with its neuroprotective function; the improvements in cognition and mood associated with BDNF would not be redundant in many peri- and postmenopausal women, presenting another potential dual benefit of irisin for postmenopausal women beyond MetS management (52).

### 2.3 Insulin resistance

Oestradiol promotes pancreatic *β*-cell survival and insulinotropism, and peripheral tissue insulin sensitisation (53, 54). Coupled with the loss of protection against VAT deposition, menopausal oestradiol depletion predisposes women to IR, which may deteriorate to T2DM in the absence of intervention. Boström et al. (13) described an anti-diabetic action for irisin in mice but did not probe the mechanistic basis for this, beyond a reduction in body fat mass secondary to elevated energy expenditure. A diverse set of insulin-sensitising actions have now been established for irisin (55). Ye et al. (56) observed irisin-induced reversal of IR in murine C2C12 cells, indicating that should the window of opportunity for prevention be missed in postmenopausal women, it may be able to reverse the IR of MetS. Irisin, like oestradiol, targets both pancreatic secretion and peripheral tissue sensitivity to improve glycaemic control.

Liu et al. (57) demonstrated improved glucose tolerance in irisin-treated rats and a dose-dependent increase in cell proliferation via the ERK/p38 MAPK pathways in INS-1 cell lines. Furthermore, irisin potentiated insulin secretion in response to high glucose media, and attenuated glucose-induced apoptosis by modulating the expression of pro- and anti-apoptotic proteins. Endogenous *Fndc5* expression in islets of Langerhans in response to high glucose was recently observed, however, expression did not correspond to augmentation of insulin content and secretion (58). Rather, irisin exerted a protective action to maintain pancreatic endocrine function under otherwise toxic hyperglycaemia—perhaps the mechanism underlying the increased insulin secretion observed by Liu et al. (57). If this occurs *in vivo*, protection against glucotoxicity, coupled with increased *β*-cell proliferative capacity could benefit postmenopausal women with early-stage glycaemic control disturbances; *β*-cell apoptosis would be limited, and regeneration promoted, thus retaining endocrine functionality and limiting progression to T2DM.

At peripheral tissues, much like oestradiol, irisin exerts insulin-like effects to reduce the IR component of MetS. Perakakis et al. (55) reviewed irisin's contributions to glucose homeostasis including a subset of irisin actions that draw parallels with oestradiol and are therefore relevant when considering postmenopausal MetS. Of note, oestradiol increases plasma membrane GLUT4 expression in adipose but is only hypothesised to do so in skeletal muscle, whereas the reverse is true of irisin, possibly reflecting dependence on local concentrations of the hormones at each tissue (59, 60). Additionally, the action of irisin at the liver presents direct mechanistic convergence with oestrogenic regulation of hepatic glucose metabolism, supporting the concept that irisin may have specific application in postmenopausal women (61, 62).

The pathogenesis of IR is multifaceted and develops against a background of wider metabolic disturbance, to which oestradiol depletion contributes. Irisin improves obesity, inflammation, and dyslipidaemia; therefore, the direct insulin-sensitising actions illustrated above exist within a broader picture of overall metabolic improvement, which likely makes a significant additional contribution to the reduction in IR.

### 2.4 Dyslipidaemia

Dyslipidaemia refers to an abnormality in plasma lipid and lipoprotein concentrations, specifically elevated low-density lipoprotein cholesterol (LDL-C) and triglycerides, and lower levels of high-density lipoprotein cholesterol (HDL-C). Dyslipidaemia promotes atherogenesis because, in simple terms, LDL-C causes blood vessel wall inflammation and atherosclerotic plaque formation, whereas HDL-C is protective against such effects (63). Menopause is associated with the acquisition of a more atherogenic blood lipid profile—a major factor in the postmenopausal increase in CVD risk (64). In a review of 295 studies into the consequences of HRT for the lipidaemic profiles of postmenopausal women, Godsland (65) concluded that all treatment regimes based on the administration of oestrogen alone were associated with elevated HDL-C and a lowering of LDL-C and total cholesterol. Oestradiol withdrawal is mechanistically implicated in the menopausal proatherogenic shift as its modulation of hepatic triglyceride and cholesterol metabolism is lost (66).

Oelmann et al. (67) reported a positive association between plasma irisin concentration and favourable plasma lipid profile (low total cholesterol, low LDL-C, high HDL-C) in men aged 40–61 years, independent of confounding factors. Whilst an equivalent association was not found in female participants, this finding in males may nevertheless provide insight into irisin's potential benefits for lipid profile against a background of low oestradiol and may therefore be relevant to the endocrinological state post-menopause. The association found in men is supported by experiments in which *Fndc5* was transgenically overexpressed in male ApoE^−/−^ mice, a model used to investigate atherogenic dyslipidaemia. Here, elevation of plasma irisin improved the lipid profiles of both chow and “Western” diet-fed animals (68).

Tang et al. (69) interrogated the molecular basis of irisin-induced improvements in lipid profile. In mice with diet-induced obesity, subcutaneous irisin infusion produced an AMPK-dependent reduction in hepatic mRNA expression and nuclear translocation of the transcription factor *Srebf2*, and a subsequent decrease in expression of its downstream targets *Hmgcr*, *Hmgcs* (encoding enzymes for cholesterol biosynthesis) and *Ldlr2* (supporting hepatocyte LDL-C uptake). Whilst effects *in vivo* were largely limited to the livers of obese mice, irisin treatment of primary hepatocytes *ex vivo* induced the same response in cells isolated from lean mice, illustrating greater potential as a therapeutic agent at supraphysiological concentrations. The downregulation of dyslipidaemia-promoting genes by irisin was more consistent in the lean-derived adipocytes, perhaps reflecting the multifactorial nature of lipid regulation under dietary stress; this raises questions about irisin's potential to reverse, as well as prevent, a postmenopausal dyslipidaemic state that often arises with the obesity component of MetS.

Excluding the LDL receptor, the expression of proteins regulating cholesterol transport in the livers of mice was largely unperturbed by irisin (69). More recently, it was proposed that irisin does in fact modulate the expression of cholesterol efflux transporters, although such observations derive from analysis of the irisin-ApoE^−/−^ mouse model, that transgenically overexpresses *Fndc5* to produce highly supraphysiological irisin levels (68). In the irisin-ApoE^−/−^ mice, mRNA and protein expression of *Abca1* and *Abcg1*, which mediate cholesterol efflux with a bias towards HDL-C biogenesis in the hepatic sinusoids, and *Abcg5* and *Abcg8*, that transport cholesterol to the bile duct for biliary excretion, were significantly increased. Furthermore, this action was conserved in enterocytes, promoting delivery of absorbed cholesterol to HDL-C in the blood or efflux back to the intestinal lumen for faecal excretion.

Early evidence demonstrates the therapeutic potential of irisin as an anti-dyslipidaemic agent. Associative studies in humans, coupled with the observation of reduced arterial atherosclerotic plaque formation in irisin-ApoE^−/−^ mice, provide preliminary but promising evidence that irisin has the therapeutic potential to improve plasma lipid profile (68). Nevertheless, it is essential to establish whether exercise-induced physiological irisin concentrations can improve cholesterol homeostasis to a degree that would be effective in targeting atherogenic dyslipidaemia as a component of MetS or if supraphysiological levels must be achieved pharmacologically. Additionally, the benefits of irisin for postmenopausal dyslipidaemia driven by oestradiol depletion require thorough investigation, possibly using ovariectomised obese mouse models, as unlike many of the parallel actions previously considered in this review, irisin appears to attenuate dyslipidaemia via distinct mechanisms that do not recapitulate the molecular actions of oestradiol.

### 2.5 Endothelial dysfunction and hypertension

Prior to menopause, blood pressure (BP) is higher in men than in women, yet in contrast, hypertension is more prevalent in postmenopausal women compared with age-matched males. Moreover, female hypertension is less well controlled by therapeutic intervention despite, on average, better adherence to BP measurement and treatment regimens (70). BP regulation is multifactorial, and the precise contribution of oestradiol is debated. Amidst the controversy, oestrogenic protection against endothelial dysfunction, a feature of MetS contributing to hypertension and CVD risk, is well established (71). Dysfunction of the vascular endothelium is characterised by reduced synthesis or bioavailability of the vasodilator nitric oxide (NO). Paracrine NO signalling is implicated in the regulation of vascular tone; therefore, dysfunction causes widespread chronic vasoconstriction, leading to hypertension.

Fu et al. (72) demonstrated that acute intravenous irisin injection produced an antihypertensive effect in male spontaneously hypertensive rats (SHR). Experiments conducted *ex vivo* on pre-constricted mesenteric arteries of the SHRs demonstrated that administration of physiological irisin concentrations increased endothelial NO synthase (NOS) phosphorylation and NO biosynthesis via an AMPK/Akt pathway but did not induce vasodilatation alone; instead, irisin enhanced the NO-dependent vasorelaxation response to acetylcholine. In contrast, irisin did not lower the BP of normotensive male Wistar-Kyoto rats. This may represent strain-specific effects, but could also reflect lower levels of cholinergic stimulation, which irisin enhances to take effect.

Furthermore, irisin attenuates free radical-mediated vascular stress to preserve endothelial function and increase NO bioavailability. In the aortas of diabetic mice, 2 weeks of daily intraperitoneal irisin injections lowered superoxide and nitrotyrosine levels, markers of oxidative and nitrative stress, respectively (73). In addition to increased phosphorylation of the constitutively expressed endothelial NOS, irisin inhibited expression of both inducible NOS, to limit NO overproduction as a driver of nitrative stress, and NADPH oxidase gp91^phox^, to attenuate superoxide production (73). Human umbilical vein endothelial cells were used to delineate the mechanism. A further endothelium-independent vasorelaxant action was observed in precontracted denuded mesentery arteries via attenuation of extracellular calcium entry into vascular smooth muscle through L-type channels, widening the scope of potential therapeutic application to individuals with pre-damaged vascular endothelia (74).

The modulation of central BP regulation by irisin is complex. Injection of recombinant irisin into the 3rd ventricle of the brain increased BP and cardiac contractility in normotensive rats, via activation of neurones in the paraventricular nucleus (75). This action is physiologically plausible—cardiac output increases during exercise to maintain BP and ensure sufficient perfusion of the muscles, and irisin secretion is induced during exercise. In contrast, intravenous irisin injection reduced BP without altering cardiac contractility, indicating a primarily vasodilatory action. In the context of exercise, this is favourable, as central sympathetic drive is sufficient to increase cardiac output, but peripheral vasodilatation, particularly at skeletal muscles where the majority of irisin is secreted, is also required. Moreover, intravenous irisin injection to SHRs reduced BP and was associated with reduced neuronal activation, oxidative stress, and inflammation at the paraventricular nucleus by an Nrf2-dependent mechanism (76). Intravenous injection protocols better mimic exercise-induced, peripheral secretion of irisin, compared with intracerebroventricular injection, and illustrate that in a more physiological scenario, irisin, or a secondary factor it regulates, is likely to exert predominantly anti-hypertensive effects at the central nervous system. Central administration likely results in a supraphysiological irisin concentration within the brain that would only be achieved (if ever) upon extreme physical exertion and almost certainly not in humans. For individuals with MetS, systemic irisin elevated by exercise will almost certainly not exert pro-hypertensive action; however, this risk should at least be considered if irisin-based pharmacological therapies are developed.

Irisin, like oestradiol, may exert modulatory action on renal fluid volume regulation (70). Zucker diabetic fatty (ZDF) rats have higher BP, impaired natriuresis and diuresis, and increased renal inflammation and angiotensin II type 1 receptor (AT_1_) expression compared with lean controls. Irisin administration to ZDF rats reduced systolic BP and improved renal function by NF-κB-dependent downregulation of AT_1_ and reduced inflammation via induction of the same anti-inflammatory cytokine profile observed in adipose tissue (77). Notably, Zucker lean rat controls did not exhibit a significant response to irisin, suggesting the anti-hypertensive action observed by Fu et al. (72) in SHRs, was not a result of strain genotype but rather improvements in renal and/or endothelial function. As hypotension is symptomatic, the lack of effect on normotensive BP would be important if administering irisin as a therapeutic agent against a subset of the conditions that form MetS, which may or may not include hypertension for a particular individual.

### 2.6 Skeletal health

Osteoporosis is the progressive loss of skeletal mass and altered structure of bone, predisposing the individual to a greater risk of fracture. Whilst osteoporosis is not a constituent feature of the MetS, it is nevertheless of significance when considering post-menopausal metabolic health, as well as the potential therapeutic impact of irisin in this context. Menopause is associated with a loss of bone mineral density and increased risk of fractures in postmenopausal women (1). Oestradiol withdrawal has been implicated as the major factor driving the loss of bone mineral density, indeed HRT therapy based on oestradiol administration (either with or without progesterone) has been associated with protection of bone mass and a lower risk of fracture (78), although administration needs to be continuous for these protective effects to persist (79). At the cellular level, osteoblasts, osteoclasts and osteocytes have all been shown to express oestrogen receptors (80–83), with bone density found to be low in both male and female oestrogen receptor knockout mice in comparison with wild-type controls (84). It has been suggested that oestrogen may exert an inhibitory effect on mature osteoclasts in adult bone, although an increase in osteoclasts following oestrogen withdrawal suggests a major impact on early differentiation with an Il6-mediated mechanism implicated (85).

In young male mice, irisin was found to increase cortical bone mass, alongside increased bone mineral density and strength (86). Moreover, irisin was found to prevent the loss of bone mass in hind limb-suspended mice (87). A recent review considered potential mechanisms underpinning the protective impact of irisin on skeletal health (88), highlighting pleiotropic effects of irisin across different bone cells which include stimulation of osteoblast differentiation and activity and a direct effect on osteocyte viability. Of note, however, irisin appears to exert two opposing effects on osteoclasts, acting to inhibit osteoclast activity indirectly via osteoprotegerin release from osteoblasts (87, 89) but also promoting osteoclast differentiation through a direct effect, with transgenic mice overexpressing *Fndc5* showing increased osteoclastogenesis and bone resorption (90). The balance between these two counteracting effects may depend on the dose and timing of irisin availability (88), and it has been postulated that these effects could collectively serve as a mechanism to maintain plasma calcium homeostasis during exercise and recovery (90).

Across a number of studies [recently reviewed by Falsetti et al. (91)] irisin levels have been found to be lowered in postmenopausal women, and across a broader range of (female and male) patients with osteoporosis irisin has often been found to correlate with bone mineral density, although this is not a consistent finding. Of particular relevance to the postmenopausal setting though, a study in OVX mice demonstrated that twice-weekly irisin administration over 5 weeks prevented the loss of trabecular bone and bone mineral density, in conjunction with greater numbers of osteoblasts (92). Collectively these studies suggest that irisin may have utility as a biomarker of postmenopausal bone health, and could have therapeutic potential.

### 2.7 Summary

**Figure 2:**
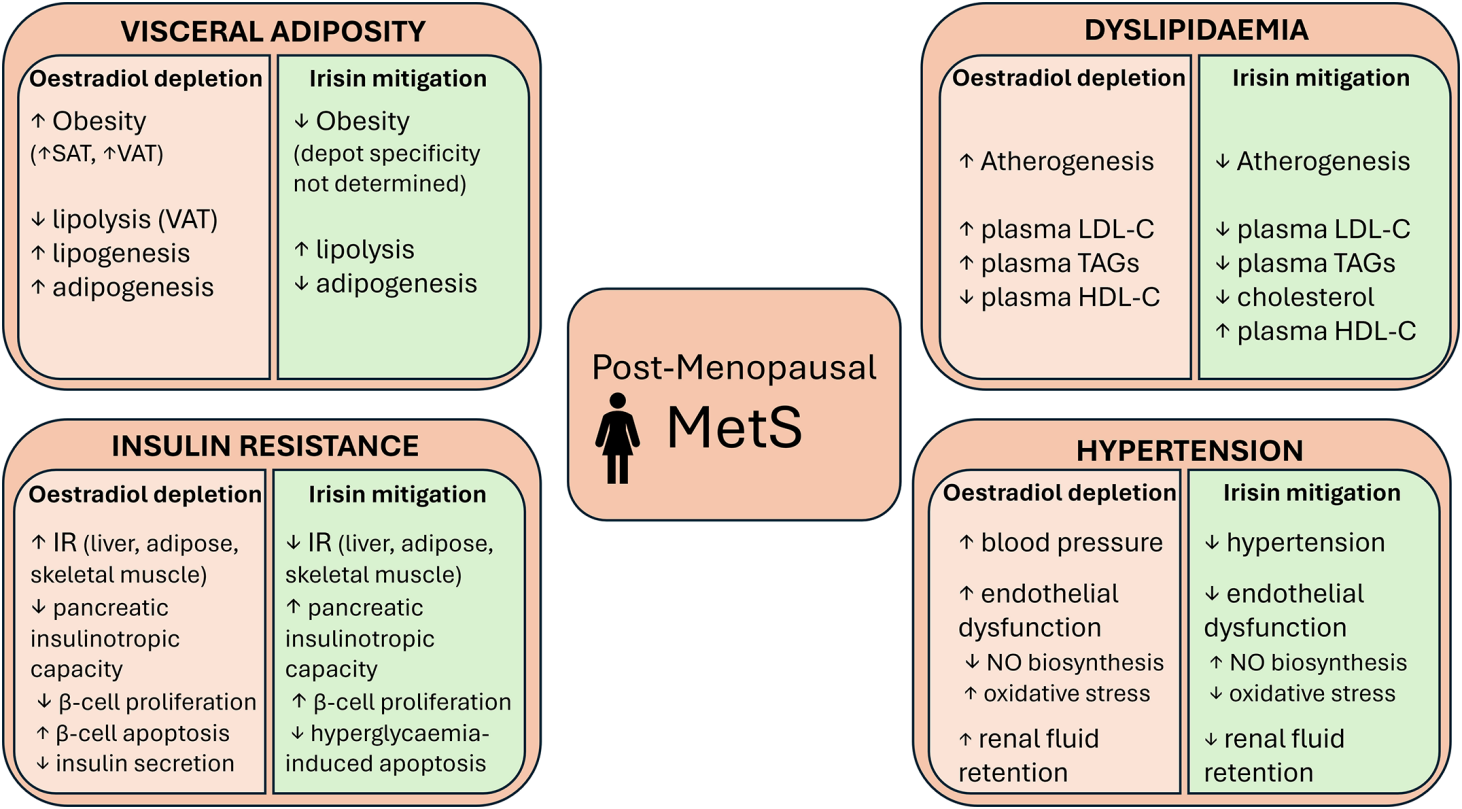
Some of the metabolic consequences of postmenopausal oestradiol depletion and the actions of irisin that could mitigate these consequences. VAT, visceral adipose tissue; SAT, subcutaneous adipose tissue; IR, insulin resistance; LDL-C, low-density lipoprotein cholesterol; HDL-C, high-density lipoprotein cholesterol; TAGs, triglycerides; NO, nitric oxide.

Taken together, there is evidence demonstrating irisin's potential to improve each condition in the MetS cluster exacerbated by menopausal oestradiol withdrawal, albeit to differing extents. Figure 2 summarises some of the metabolic consequences of oestradiol depletion and potential irisin-mediated mitigations.

## 3 Discussion

### 3.1 Integrated aspects of irisin's action and therapeutic potential

A rat model generated by combining ovariectomy and a HFD has been used to investigate the specific potential of irisin as a therapeutic agent for postmenopausal MetS (93). Daily injections of irisin over 8 weeks ameliorated development of the OVX/HFD-induced MetS phenotype, including improvements in obesity, energy expenditure, insulin sensitivity and lipid profile in comparison with OVX/HFD controls. The study therefore presents a compelling case that irisin may be a viable pharmacological agent for targeting MetS in postmenopausal women, and highlights the need for more focused, condition-specific research to evaluate therapeutic potential in humans.

Multiple studies have sought to analyse the effects of different exercise modalities and intensities on plasma irisin concentrations and metabolic improvements in humans, but results lack here consensus. A selection of studies, specifically in women, are presented in Table 2 (22, 37, 94–97). The greatest challenge to understanding the action of exercise-induced irisin in humans is delineating the relative contribution of irisin to any metabolic improvements from that of the increased energy expenditure as a result of exercise itself. Intravenous irisin infusion experiments may be required to provide a definitive answer although none have been performed to date.

**Table 2:** A selection of studies that have investigated the effect of exercise on plasma irisin concentrations and metabolic improvements (in long-term studies only).

Reference	Subjects	Blood sample	Exercise modality/intensity	Plasma irisin concentration	MetS improvement
Long-term exercise regimens					
(94)	Obese, young women	48 h after final session	Pilates (3 times per week, 8 weeks)	Increase	Reduced insulin resistance
			Total body resistance training (3 times per week, 8 weeks)	Increase	Reduced insulin resistance
(37)	Postmenopausal women	24 h after final session	High-intensity concurrent interval training (3 times per week, 10 weeks)	Increase	Reduced insulin resistance, SAT, VAT, and total abdominal fat mass
			Moderate-intensity interval training (3 times per week, 10 weeks)	No change	Reduced insulin resistance and total abdominal fat mass
(22)	Postmenopausal women	36 h after final session	Combined aerobic and MSROM training (3 times per week, 8 weeks)	Increase	Reduced insulin resistance, improved lipid profile
(95)	Women with MetS (40–60 years)	24 h after final session	Aerobic exercise (3 times per week, 8 weeks)	No change	Reduced obesity, insulin resistance, improved lipid profile
			Resistance training (2–3 times per week, 8 weeks)	No change	Reduced obesity and insulin resistance, improved lipid profile
			Combined training (2–3 times per week, 8 weeks)	No change	Reduced obesity and insulin resistance, improved lipid profile
Acute exercise					
(96)	Obese, young women	During and after exercise	Moderate intensity exercise (isolated session)	Increase (sustained for at least 190 min)	n/a
			High intensity exercise (isolated session)	Increase (during exercise only)	
(97)	Young women	During and after exercise	Prolonged, moderate aerobic exercise	Increase (peak mid exercise, then decline)	n/a

MSORM, muscular strength range of movement.

Experiments analysing acute bouts of exercise have shown transient irisin elevations during activity and this may explain the heterogeneity in results from long-term studies. Where no change in plasma irisin is reported following regular exercise, it cannot be definitively concluded that irisin played no role in the observed improvements in MetS, as blood plasma measurements occurred over 24 h after the final session (37, 95). The dynamics of irisin release and clearance during exercise lack clarity, as does the physiological significance of basal levels vs. transients. It is possible that transients reflect FNDC5 depletion due to cleavage outstripping translation and/or membrane trafficking during intense exercise. More moderate activity and regular exercise might therefore increase basal *FNDC5* expression, and therefore overall secretory capacity, facilitating an increase in basal plasma irisin. Despite the uncertainty surrounding irisin's specific contribution to MetS mitigation, it is encouraging that multiple exercise modalities have been shown to increase basal levels, and higher intensity exercise broadly confers a larger increase in these basal levels.

### 3.2 Outstanding questions and future directions

Despite evidence for a mechanistic basis, there remain major challenges to the hypothesis that irisin could mitigate MetS. Most mechanistic evidence is derived from cell line or rodent studies. Rodent and human irisin are homologous peptides but cannot be assumed to exert equivalent actions across physiologically distinct species. Where human studies have taken place, there has been minimal scope to interrogate molecular mechanisms and confounding variables cannot be controlled, leading to weak associative conclusions. Most significantly, a comparison of physiological and experimental concentrations of irisin challenges the evidence accumulated to date and its relevance to humans, owing to the supraphysiological levels used in most experiments. Tandem mass spectroscopy data suggested the concentration of human plasma irisin, as a 25 kDa bioactive glycosylated dimer, is in the range of 3–5 ng/ml, depending on activity status in adult males, whereas in rats, concentrations in the range of 300–600 ng/ml have been reported (16, 17, 72). Determining the full range of physiological irisin concentrations across the human population, with varied representations of age, weight, sex, and ethnicity, is critical to establishing the utility of exercise-based therapies, alongside informing future experimental protocols.

Further challenges arise when considering irisin and its actions specifically against a background of postmenopausal MetS. In one study (97), young women were stratified by menstrual cycle stage. No difference was found in the amplitude of exercise-induced irisin elevations between the early follicular and mid-luteal phases, suggesting oestradiol availability, at least in young women, does not impact on irisin secretion during exercise. Conversely, skeletal muscle is the main source of irisin in humans, yet menopause accelerates the development of sarcopenia; whilst oestradiol level may not alter irisin secretion, physical capacity to do so may decline (98). This may be remedied with exercise, which alongside stimulating *FNDC5* expression, prevents the decline in muscle mass, however, still may not result in effective concentrations.

Despite some inconsistencies in the literature, several studies have shown basal irisin concentration is positively associated with obesity, a paradoxical observation as overweight individuals typically exercise less (99). Park et al. (100) found higher irisin levels in subjects with MetS than metabolically healthy subjects, and identified positive associations between plasma irisin concentration and BMI, BP, IR, and blood lipid profile. The greater adipose mass may itself be a potential source of the elevated irisin although adipose is normally considered to make a minor contribution to circulating levels. Alternatively, the authors suggest that the elevated irisin level may act to compensate for irisin resistance. Any therapeutic applications of irisin would be significantly limited by the development or pre-existence of irisin resistance and this deserves further consideration.

It is likely that in humans the irisin concentration required to mitigate MetS cannot be achieved via exercise alone. The concept of an injectable form of irisin is not implausible and was alluded to by Boström et al. (13) at the time of irisin's discovery. Injections of recombinant irisin, or a modified version of the peptide, would increase accessibility to irisin therapies, especially for those with later-stage MetS causing reduced mobility. Additional exercise-independent methods of stimulating irisin secretion may also be considered, and for example, a regular weekly thermotherapy regimen of intermittent heat exposure by bathing increased plasma irisin and basal metabolic rate, and decreased waist circumference and body fat percentage in postmenopausal women (101). Furthermore, some existing drugs targeting component conditions of MetS, including metformin, simvastatin and exenatide, may upregulate skeletal muscle *FNDC5* expression and/or increase plasma irisin concentrations (102). Alternatively, inorganic nitrate administration enhanced *Fndc5*/irisin expression in C2C12 myotubes and, when delivered orally at a moderate dose, in the soleus and gastrocnemius muscle of rats, alongside enhanced plasma irisin levels (103). Intriguingly, a similar elevation in plasma irisin was seen in human volunteers consuming nitrate-containing beetroot juice over a 7-day period (103) suggesting that exercise mimetics such as nitrate may provide a route to elevate irisin.

Irisin's apparent therapeutic potential is of growing interest but remains hypothetical due to a lack of clarity surrounding fundamental elements of its biology. The precise mechanism of action needs to be determined, from identifying the receptor(s), which itself might be targeted independently of irisin, to determining intracellular pathways activated in a tissue-specific manner. Currently, these uncertainties place significant limitations on progression. The proteases that cleave irisin from FNDC5 are also unidentified, and it is unclear whether *FNDC5* expression or proteolytic cleavage is the rate-limiting step in irisin secretion. The impact of glycosylation and dimerisation on biological action is also undetermined. From a clinical perspective, the therapeutic window must be established from dose-response studies. Unanswered questions of relevance to irisin's basic science and therapeutic potential specifically in postmenopausal women with MetS remain. To begin to address these, any sexually dimorphic effects or interactions with oestrogens should be identified.

## 4 Conclusion

Postmenopausal MetS is clinically neglected and considered part of the natural ageing process despite its consequences for quality of life and risk of progression to more severe diseases. There is strong *in vitro* evidence that irisin could mitigate each component condition of MetS, with many of these actions confirmed *in vivo,* albeit in rodents. It is rare that a relatively novel agent with such potential for safe, inexpensive, and accessible therapeutic application is identified. Twelve years on from discovery, the actions of irisin, particularly on metabolic health, are reasonably well characterised. The focus of research is beginning to shift from defining biological actions and elucidating their molecular basis to understanding how the pleiotropic effects of this novel hormone can be exploited in clinical practice to treat postmenopausal MetS, and a multitude of other conditions. Although still a distant prospect, this research may provide a comprehensive treatment of a condition that, for too long, has been overlooked.
